# Open discectomy vs microdiscectomy for lumbar disc herniation - a protocol for ****a pragmatic comparative effectiveness study

**DOI:** 10.12688/f1000research.9015.1

**Published:** 2016-09-02

**Authors:** Andreas Sørlie, Sasha Gulati, Charalampis Giannadakis, Sven M. Carlsen, Øyvind Salvesen, Øystein P. Nygaard, Tore K. Solberg

**Affiliations:** 1 The Norwegian National Registry for Spine Surgery (NORspine), University Hospital of North Norway (UNN), Tromsø, Norway; 2 Department of Neurosurgery, University Hospital of North Norway (UNN), Tromsø, Norway; 3 Department of Neurosurgery, St. Olavs University Hospital, Trondheim, Norway; 4 National Advisory Unit on Spinal Surgery, Norwegian University of Science and Technology (NTNU), Trondheim, Norway; 5 Department of Neuroscience, Norwegian University of Science and Technology (NTNU), Trondheim, Norway; 6 Department of Cancer Research and Molecular Medicine, Norwegian University of Science and Technology (NTNU), Trondheim, Norway; 7 Department of Endocrinology, St. Olavs University Hospital, Trondheim, Norway; 8 Department of Clinical Medicine, University of Tromsø, Tromsø, Norway

**Keywords:** Microdiscectomy, Discectomy, multicenter, comparative, Effectiveness, Registry, pragmatic (study protocol), Propensity match

## Abstract

**Introduction**:

Since the introduction of lumbar microdiscectomy in the 1970’s, many studies have attempted to compare the effectiveness of this method with that of standard open discectomy with conflicting results. This observational study is designed to compare the relative effectiveness of microdiscectomy (MD) with open discectomy (OD) for treating lumbar disc herniation, -within a large cohort, recruited from daily clinical practice.

**Methods and analysis: **

This study will include patients registered in the Norwegian Registry for Spine Surgery (NORspine). This clinical registry collects prospective data, including preoperative and postoperative outcome measures as well as individual and demographic parameters. The primary outcome is change in Oswestry disability index between baseline and 12 months after surgery. Secondary outcome measures are improvement of leg pain and changes in health related quality of life measured by the Euro-Qol-5D between baseline and 12 months after surgery, complications to surgery, duration of surgical procedures and length of hospital stay.

## Background

Lumbar disc herniation is a common cause of sciatic pain and functional disability. Although most patients are relieved from their symptoms without surgical treatment, there is consensus for operating on selected patients with persistent radicular pain after 2–6 months ( Atlas *et al.*, 927–35; Schoenfeld & Bono, 1963–70; Weinstein *et al.*, 2789–800). Surgical discectomy gives earlier relief of symptoms, enabling patients to return to their work and other daily activities more rapidly. In cases where the pain is incapacitating and the patient is bedridden with strong pain medication or in the cases of progressive paresis, there are sometimes indications for earlier surgical intervention. The aim is to relieve the patient of the pain and to prevent late sequela, like permanent paresis and neuropathic pain. Moreover, prolonged sick leave may lead to undesirable lifestyle changes and reduce motivation to return to work ( Sieurin *et al.*, 50–56).

In 1977, Yasargil and Caspar independently introduced the technique of microdiscectomy for treating lumbar disc herniation ( Yasargil, 81) ( Caspar *et al.*, 78–86). This technique offers better visual control of the operation field, through less traumatic and smaller incisions, compared to the standard open discectomy. The role of other treatment options such as minimally invasive discectomy (MID), chemonucleolysis and endoscopic discectomy is still unclear ( Rasouli *et al.*, CD010328) ( Gibson & Waddell, 1735–47) and both open discectomy and microdiscectomy are still considered the best surgical treatment options ( Gibson & Waddell, 1735–47) and are the most commonly used treatment modalities today.

Previous randomized trials have been small single centre studies and have been unable to demonstrate any difference between the two treatment modalities ( Katayama *et al.*, 344–47; Tullberg *et al.*, 24–27). In one recent prospective non-randomised multicenter trial of 261 patients, they found significant better improvement of radicular pain at 12 months follow up after open discectomy in comparison to microdiscectomy. For all other outcome parameters, there were no significant differences between the groups ( Porchet *et al.*, 360–66). However, the two treatment cohorts were not matched and uneven with respect to risk factors that might influence the outcome. A systematic review done by Gibson and Waddell in 2007, found no significant difference in outcome between the two treatment modalities and they concluded that even though open discectomy remains the “standard”, further studies comparing these two surgical methods are warranted. ( Gibson & Waddell, 1735–47).

The current study is a large multicenter observational study comparing the relative effectiveness of the two treatments. Data are obtained as part of daily clinical practice at several institutions, resulting in a high external validity. Moreover, the size of the study allows for propensity matching, making the two groups comparable in most aspects for a close approximation to a randomized controlled trial.

### Definition of terms

This observational study is designed to compare the relative effectiveness of discectomy with or without visual enhancement for treating lumbar disc herniation. We will use the term “open discectomy” for discectomy done without the use of visual enhancement, while the term “microdiscectomy” entails the use of visual enhancement like a microscope or loupes.

There are some variations in the surgical procedure, depending on peroperative findings as well as surgeon preferences. One such variation is whether the disc space is entered during the operation. In this study, the term discectomy includes procedures were sequestrectomy is done with or without entering the disc space. Variations of the surgical procedure (in both groups), such as discectomy vs. sequestrectomy and concomitant decompression of the nerve root due to recess- or foraminal stenosis will be reported (See Table 2 in Supplementary material).

## Aims of the study

The primary aim of this study is to compare the effectiveness of discectomy with or without visual enhancement (i.e. microdiscectomy vs. open discectomy) for treating lumbar disc herniation.

## Methods and materials

**Study population:** Data for this cohort study have been collected through NORspine which is a Norwegian national comprehensive clinical registry for quality control and research of surgical intervention in the spine. Participation in the registration is voluntary, however patients are recommended to participate for contributing to the completeness of the registry. Patients receive the same treatment irrespective of their participation in the registry.

Patients operated on between October 2006 and the end of May 2014 will be screened for study eligibility. In the registry, the follow-up time of the operation (at baseline) is 3 and 12 months.

**Inclusion criteria:**

1. Included in the NORspine registry
2. Surgery for herniated lumbar disc disease using open discectomy or microdiscectomy with preservation of midline structures (spinous process and ligaments).

**Exclusion criteria:**

1. Operated in > 1 level.
2. Previous operations in the lumbar spine.
3. Patients with deformities in the lumbar spine (spondylolisthesis or scoliosis).
4. If intervention included more comprehensive surgery like laminectomy or fusion.
5. Other minimally invasive procedures.
6. Far lateral approaches for lateral disc herniations.

### Data collection and registration by the NORspine registry

**Figure 1.**
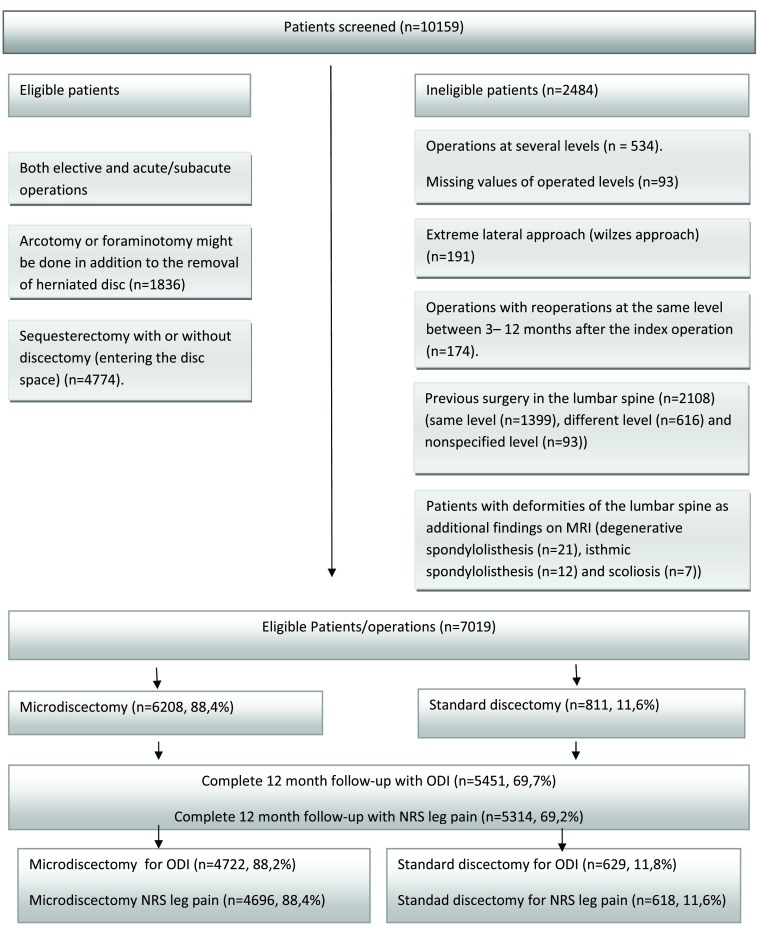
Flow diagram of patient enrollment and follow up. N refers to surgical procedures.

On admission for surgery, patients complete the baseline questionnaire, which includes questions about demographic and lifestyle issues in addition to the outcome measures and duration of symptoms. Information about marital status, educational level, employment status, body mass index, and tobacco-smoking is available in the NORspine registry. The surgeon records data concerning diagnosis, comorbidity, *American Society of Anesthesiologists (ASA)* grade, image findings, treatment, use of prophylactic antibiotics and peroperative complications. Duration of the surgical procedure and hospital stay are recorded by hospital staff (trained nurse or health secretary). A questionnaire is distributed by regular mail 3 and 12 months after surgery, completed at home by the patient and returned to the central registry unit of the NORspine, without involving the treating hospitals. According to a standardized set of questions patients report postoperative complications having occurred within 3 months of follow-up. Non respondents receive one reminder with a new copy of the questionnaire. The response rates to the registry after 1 year for the relevant group of patients is around 70% ( Figure 1). Figure 1 shows the patient population before the statistical analysis will commence.

**Ethics and dissemination:** NORspine has been evaluated and approved by the Norwegian Data Protection Authority. All participants have provided written informed consent that the data collected in NORspine can be used for research purposes and the results will be disseminated through peer-reviewed publications. The study has been approved by the regional committee for medical and health research in central Norway (REK central) 2016/840.

**Primary outcome measures:** The primary outcome measure is change in functional outcome defined by Oswestry disability index (ODI) between baseline and follow-up at 12 months (mean change of ODI and proportion of patients achieving the minimally clinical important difference (MCID) between the two groups).

The functional outcome is measured with V.2.0 of ODI and translated into Norwegian and tested for psychometric properties as outlined by Grotle *et al.* ( Grotle *et al.*, 241–47). ODI is one of the principal condition-specific outcome measures used in the management of spinal disorders. ODI contains 10 questions on limitation of activities of daily living. Each variable is rated on a 0 – 5 point scale, summarised and converted into a percentage score, ranging from 0 to 100 (0= no disability).

There are great variations in the estimated values for MCID after spine surgery and the value ranges from 8 – 15 in the literature ( Hellum *et al.*, d2786; Nerland *et al.*, h1603; Ostelo *et al.*, 90–94). MCID is by the definition the minimal value of improvement in a measurement that exceeds the normal statistical variation and also is experienced by the patient as a definite improvement. In most cases the value of MCID does not however represent the desired effect of the treatment, i.e. it is not a criterium for success. Solberg *et al.* established anchor based success criteria ( Solberg *et al.*, 196–201) after discectomy as change in ODI ≥ 20 and change in NRS leg pain ≥3,5. Both MCID and cutoff values for success are useful tools for evaluating patient outcome and can be used to compare proportions of patients responding to the treatment. To estimate the proportion of responders to the treatment, we define an improvement of ODI ≥ 10 and NRS leg pain ≥ 2 as cutoff of minimally important clinical differences ( Ostelo *et al.*, 90–94) ( Nerland *et al.*, h1603) ( Brox *et al.*, 145–55) ( Hellum *et al.*, d2786) .

**Secondary outcome measures:** Secondary measures are:

1. Mean changes in health related quality of life measured with the EQ-5D between baseline and 12 months follow-up.
2. Mean improvement of NRS leg pain between baseline and follow-up at 12 months.
3. Mean improvement in back pain using the Numeric Rating Scale (NRS).
4. Duration of the surgical procedure.
5. Duration of hospital stay.
6. Perioperative complications.
7. Postoperative complications.

EQ-5D is a generic and preference-weighted measure of HRQL. The Norwegian version of EQ-5D has shown good psychometric properties and has been validated for patient populations similar to that in our study ( Solberg *et al.*, 1000–07). EQ-5D evaluates five dimensions: mobility, self-care, activities of daily living, pain, and anxiety and/or depression. For each dimension, the patient describes three possible levels of problems (non, mild-to-moderate and severe). This descriptive system therefore contains 243 (3 ^5^) combinations or index values for health status. EQ-5D total score ranges from -0.6 to 1, where 1 corresponds to perfect health and negative values are considered worse than death ( Dolan *et al.*, 141–54).

Leg pain will be assessed by the Numeric Rating Scale (NRS), ranging from 0–10, where 0 = no pain and 10 = worst pain.

Perioperative complications are reported at the time of inclusion (immediately after surgery) and include dural tear, nerve root injury, bleeding requiring transfusion, respiratory or cardiovascular complications and anaphylaxis.

Postoperative complications are registered by the patient on the follow up questionnaire after 3 months and includes wound infection, deep venous thrombosis, pulmonary embolism, pneumonia and urinary tract infection. We also define reoperation within 3 months as a complication.

## Surgical procedures

Since this is a multicenter trial, variations in the surgical management and the surgical procedures can only be described in general terms and in accordance with the data collected in the NORspine registry. The microsurgical discectomy is well described and involves preoperative fluoroscopy for detection of the target level, paramedian or median incision of about 3–6 cm, straight or curved opening of the paravertebral muscular fascia, subperiosteal release of the paravertebral musculature from the spinous process and basal lamina above and below the target disc-level. Caspar self-retaining retractors and a microscope or loupes are introduced. In most cases a flavectomy and arcotomy of the lamina above the disc-level is done. Careful mobilization of the dural sac and the nerve-root medially, before evacuating the herniated disc. This might involve entering the disc space, or just removing a free sequestrated disc fragment (sequestrectomy). The traditional open discectomy did not involve retractors which minimizes the incision to unilateral muscular dissection. However, many institutions that perform standard open discectomy also use equivalents to the caspar retractors. In which case the procedure is in principle the same as described for microdiscectomy, except regarding the use of microscope or other visually enhancing tools (like loupes) and may require a larger incision and more soft tissue damage.

## Statistical analyses

This study will test the equivalence of the clinical effectiveness of the two surgical techniques. Case analysis will be done using mixed linear model analysis in both the aggregate cohort and a propensity matched cohort. The minimal clinical important difference for change in the mean ODI score is considered to be in the range of 10 points. If mean changes of ODI is < 10, the treatments are considered equal with respect to effectiveness. Since there is no clear consensus on how large the MCID should be between two treatment groups and as the MCID also describes effects of interventions on an individual level, we would like to present the proportion of patients having MCID as result of the treatment – in both the aggregate cohort and the propensity matched cohort. In the analysis of primary and secondary outcome measures, adjustments for age, body mass index, and preoperative ODI, as well as smoking habits will be done. Statistical significance level is defined as p <0.05 with no adjustments made for multiple comparisons. Baseline and follow-up measurements will be assumed to be normally distributed provided this assumption is confirmed by Q-Q plots. To evaluate the magnitude of change in EQ-5D score, we will estimate effect sizes according to the method of Kazis. ( Kazis *et al.*, S178–S189). An effect size of 0.8 or more is considered to be large. In the mixed model patients will not be excluded from the analysis, if the variable is missing at some (but not all) time points after baseline. This strategy is in line with a study showing that it is not necessary to handle missing data using multiple imputations before performing a mixed model analysis on longitudinal data.( Twisk *et al.*, 1022–28). In the additional analyses (categorical data at three months’ follow-up), we will not replace missing data. Continuous variables will be analysed using an unpaired two tailed t test for normally distributed data, and Mann- Whitney U test for skewed distribution. A X ^2^ analysis will be used to compare discrete variables. The content of tables and figures (see Supplementary material) are predefined before the statistical analyses are done, and no information will be deleted when results are known. We do not plan any additional exploratory statistical analyses.

To achieve the closest approximate to a randomized clinical trial, we will use matching approach technique of using propensity scores, as opposed to stratification or regression adjustment. It provides the greatest balance between the two treatment groups ( Hemmila *et al.*, 939–45) ( Austin *et al.*, 734–53). We will generate propensity scores for surgical technique using logistic regression and adjusting for baseline covariates that could influence treatment outcomes, including age, sex, life partner, comorbidity, body mass index, smoking, educational level, and preoperative ODI score. All covariates are entered into a logistic regression analysis, and we will fit a maximum likelihood model based on these covariates as predictors of surgical technique. The coefficients for these predictors of surgical technique are used to calculate a propensity score of 0 to 1 for each patient. Based on the calculated propensity scores, two evenly matched groups will be formed for surgical technique using a matching algorithm with the common caliper set at 0.010. This dataset will be referred to as the “propensity matched cohort.” We will analyze continuous variables using a related samples two tailed *t* test for data with a normal distribution and continuous data exhibiting a skewed distribution using the Wilcoxon matched pair signed rank test. We will use the McNemar’s test to compare discrete variables.

## Study limitations

The main limitation of this study, as its purpose is to compare two different treatment modalities, is that it is not a randomized trial. Rather than comparing the efficacy of the treatment, we will focus on the effectiveness, and the findings might entail other treatment related differences than that of the surgical procedure alone. However, the use of propensity matched groups will minimize these potential differences.

For the standard discectomies, we cannot differentiate between operations done with unilateral or bilateral muscular dissection.

The inclusion rate to the NORspine registry for lumbar herniated disc procedures is around 65%. The inclusion rate is closely monitored through comparing registered patients with data from the Norwegian Patient Registry (NPR) (where all patients operations are registered for performance-based financing). Loss to follow up may be approximately 30%. However, a previous study has shown that nonresponders have the same outcome after surgery as those who respond to the follow up questionnaires ( Solberg *et al.*, 56–63). These factors might nevertheless limit the validity of our findings.

## Conclusion

This is a protocol for an observational study designed to compare the relative effectiveness of microdiscectomy with open discectomy for treating lumbar disc herniation. The study is based on data from the NORspine registry collected from 30 different institutions in Norway from October 2006 – 12. May 2014. We have discussed the details of the clinical registry and patient enrolment as well as the planned statistical analysis.

## Supplementary material

**Table 1: Baseline characteristics for both treatment groups.**

.

**Table 2: Procedural differences and differences in peroperative- and postoperative complications between the two groups.**

.

**Table 3: Changes in ODI and EQ-5D between baseline and one year after the operation within each treatment group for both the aggregate cohort and the matched cohort.**

.

**Table 4: Complete case analyses of categorical outcome measures in the aggregate cohort and propensity matched cohort. MCID is defined as dODI ≥ 10 and dNRS ≥ 2.**
