# Supplementary material for: Open discectomy vs microdiscectomy for lumbar disc herniation - a protocol for a pragmatic comparative effectiveness study
Source: F1000Res. 2016 Sep 2;5:2170. [Version 1] doi: 10.12688/f1000research.9015.1 (PMC5089132; doi:10.12688/f1000research.9015.1)
Supplement: Supplementary file 1 [file f1000research-5-9699-s0000.tgz › ac9cc4bc-0489-421a-b950-464054f70252.docx]

|  | **Aggregate Cohort** | |  |  | **Propensity-Matched Cohort** | |  |
| --- | --- | --- | --- | --- | --- | --- | --- |
| **Variable** | **Standard Discectomy group** | **Micro-discectomy group** | **P-value** |  | **Standard Discectomy group** | **Micro-discectomy group** | **P- value** |
| No (%) |  |  |  |  |  |  |  |
| Age – yr |  |  |  |  |  |  |  |
| Female sex – no. (%) |  |  |  |  |  |  |  |
| Married or partner – no. (%) |  |  |  |  |  |  |  |
| Attended college – no. (%) |  |  |  |  |  |  |  |
| Body Mass Index |  |  |  |  |  |  |  |
| Current smoker – no. (%) |  |  |  |  |  |  |  |
| Comorbidity – no. (%) |  |  |  |  |  |  |  |
| ASA grade > 2 |  |  |  |  |  |  |  |
| Preoperative ODI |  |  |  |  |  |  |  |
| Preoperative EQ-5D |  |  |  |  |  |  |  |
| Preoperative Leg pain (NRS) |  |  |  |  |  |  |  |
| Preoperative Back pain (NRS) |  |  |  |  |  |  |  |
| Duration of leg pain > 1Yr - No (%) |  |  |  |  |  |  |  |
| Preoperative MRI – No (%) |  |  |  |  |  |  |  |
| Preoperative CT – No (%) |  |  |  |  |  |  |  |
| Receiving Sick leave benefit (%) |  |  |  |  |  |  |  |
| Using pain medication - No (%) |  |  |  |  |  |  |  |
|  |  |  |  |  |  |  |  |
|  |  |  |  |  |  |  |  |
|  |  |  |  |  |  |  |  |

Table 1: Baseline characteristics for both treatment groups.
