# Supplementary material for: Open discectomy vs microdiscectomy for lumbar disc herniation - a protocol for a pragmatic comparative effectiveness study
Source: F1000Res. 2016 Sep 2;5:2170. [Version 1] doi: 10.12688/f1000research.9015.1 (PMC5089132; doi:10.12688/f1000research.9015.1)
Supplement: Supplementary file 2 [file f1000research-5-9699-s0001.tgz › fba1a445-03d0-42c2-a392-32052d5a0735.docx]

|  | **Aggregate Cohort (n=)** | | | |  | **Propensity-Matched Cohort (n=)** | | | |
| --- | --- | --- | --- | --- | --- | --- | --- | --- | --- |
| **Variable** | **Standard discectomy group** | **Micro- discectomy group** | **Difference (95% CI)** | **P-value** |  | **Standard discectomy group** | **Micro- discectomy group** | **Difference (95% CI)** | **P- value** |
| **Operated in level L2-L3** |  |  |  |  |  |  |  |  |  |
| **Operated in level L3-L4** |  |  |  |  |  |  |  |  |  |
| **Operated in level L4-L5** |  |  |  |  |  |  |  |  |  |
| **Operated in level L5-S1** |  |  |  |  |  |  |  |  |  |
| **Prophylactic antibiotic treatment - No(%)** |  |  |  |  |  |  |  |  |  |
| **Operation time**  **(minutes)** |  |  |  |  |  |  |  |  |  |
| **Concomittant Discectomy (entering the disc space)** |  |  |  |  |  |  |  |  |  |
| **Concomittant decompression of of foraminal /recess stenoses at same level** |  |  |  |  |  |  |  |  |  |
| **Days in hospital no.** |  |  |  |  |  |  |  |  |  |
| **Postoperative Drain – No**  **(%)** |  |  |  |  |  |  |  |  |  |
| **Patients with complications**  **-no. (%)** |  |  |  |  |  |  |  |  |  |
| **Perioperative**  **complications -no. (%)** |  |  |  |  |  |  |  |  |  |
| - Dural tear or  spinal fluid leak |  |  |  |  |  |  |  |  |  |
| - Nerve injury |  |  |  |  |  |  |  |  |  |
| - Blood replacement  or postoperative  hematoma |  |  |  |  |  |  |  |  |  |
| **-** Cardiovascular  complications |  |  |  |  |  |  |  |  |  |
| **-** Respiratory  complications |  |  |  |  |  |  |  |  |  |
| - Anaphylactic  reaction |  |  |  |  |  |  |  |  |  |
| **-** Wrong level surgery |  |  |  |  |  |  |  |  |  |
| **Complications within**  **3 months**  **-no. (%)** |  |  |  |  |  |  |  |  |  |
| - Wound infection |  |  |  |  |  |  |  |  |  |
| - Urinary tract  infection |  |  |  |  |  |  |  |  |  |
| - Micturition problems |  |  |  |  |  |  |  |  |  |
| - Pneumonia |  |  |  |  |  |  |  |  |  |
| - Pulmonary  embolism |  |  |  |  |  |  |  |  |  |
| - Deep venous  thrombosis |  |  |  |  |  |  |  |  |  |
| **Reoperated within 90 days** |  |  |  |  |  |  |  |  |  |

# Table 2: Procedural differences and differences in peroperative- and postoperative complications between the two groups.
