# Supplementary material for: Open discectomy vs microdiscectomy for lumbar disc herniation - a protocol for a pragmatic comparative effectiveness study
Source: F1000Res. 2016 Sep 2;5:2170. [Version 1] doi: 10.12688/f1000research.9015.1 (PMC5089132; doi:10.12688/f1000research.9015.1)
Supplement: Supplementary file 3 [file f1000research-5-9699-s0002.tgz › 5bff9174-2366-4b61-96e2-2c50dcb6052c.docx]

|  | **Complete case analysis** | | | | | | | |  | **Mixed linear model analysis** | | | | | | | |
| --- | --- | --- | --- | --- | --- | --- | --- | --- | --- | --- | --- | --- | --- | --- | --- | --- | --- |
|  | **Standard discectomy** | | | **Microdiscectomy** | | | **Difference in mean change between groups (95% CI)** | **P for equivalence** |  | **Standard discectomy** | | | **Microdiscectomy** | | | **Difference in mean change between groups (95% CI)** | **P for equivalence** |
|  | **Baseline** | **One year** | **Mean change** | **Baseline** | **One year** | **Mean change** |  |  |  | **Baseline** | **One year** | **Mean change** | **Baseline** | **One year** | **Mean change** |  |  |
| **Aggregate cohort** |  |  |  |  |  |  |  |  |  |  |  |  |  |  |  |  |  |
| *ODI* |  |  |  |  |  |  |  |  |  |  |  |  |  |  |  |  |  |
| *EQ-5D* |  |  |  |  |  |  |  |  |  |  |  |  |  |  |  |  |  |
| **Matched cohort** |  | | |  | | |  |  |  |  | | |  | | |  |  |
| *ODI* |  |  |  |  |  |  |  |  |  |  |  |  |  |  |  |  |  |
| *EQ-5D* |  |  |  |  |  |  |  |  |  |  |  |  |  |  |  |  |  |

# Table 3; Changes in ODI and EQ-5D between baseline and one year after the operation within each treatment group for both the aggregate cohort and the matched cohort.
