# Supplementary material for: Open discectomy vs microdiscectomy for lumbar disc herniation - a protocol for a pragmatic comparative effectiveness study
Source: F1000Res. 2016 Sep 2;5:2170. [Version 1] doi: 10.12688/f1000research.9015.1 (PMC5089132; doi:10.12688/f1000research.9015.1)
Supplement: Supplementary file 4 [file f1000research-5-9699-s0003.tgz › fffc3c18-fa59-4011-b56d-25483c2f59f9.docx]

|  | **Aggregate Cohort (n=)** | | | |  | **Propensity-Matched Cohort (n=)** | | | |
| --- | --- | --- | --- | --- | --- | --- | --- | --- | --- |
| **Variable** | **Standard discectomy group** | **Micro- discectomy group** | **Difference (95% CI)** | **P-value** |  | **Standard discectomy group** | **Micro- discectomy group** | **Difference (95% CI)** | **P- value** |
| **Proportion of patients with MCID of ODI – No (%)** |  |  |  |  |  |  |  |  |  |
| **Proportion of patients with MCID of Leg NRS – No (%)** |  |  |  |  |  |  |  |  |  |

Table 4: Complete case analyses of categorical outcome measures in the aggregate cohort and propensity matched cohort. MCID is defined as dODI ≥ 10 and dNRS ≥ 2.
