# Supplementary material for: Open discectomy vs microdiscectomy for lumbar disc herniation - a protocol for a pragmatic comparative effectiveness study
Source: F1000Res. 2016 Sep 2;5:2170. [Version 1] doi: 10.12688/f1000research.9015.1 (PMC5089132; doi:10.12688/f1000research.9015.1)
Supplement: Peer review history

# Peer review: Referee response for version 1

**DOI:** 10.5256/f1000research.9699.r17020

**Published:** 2016-10-17

**Authors:** Edilson Forlin

**Affiliations:** 1 Department of Orthopedics, Hospital Pequeno Principe, Curitiba, Parana, 80250-060, Brazil

I believe the protocol presented is interesting and will be able to provide important information on which technique may have advantages to address discal herniation with surgical indication. The objectives and assessment criteria are clear and consistent. The assessment covers the main points of clinical practice when we think of the results.

 The limitations and difficulties of the study are not small. The lack of control over information and aspects of surgery as pointed out by the authors can actually hinder the interpretation of results. It would be interesting to have a profile on surgeons in both groups. It seems to me microdiscectomy is preferred by younger surgeons. This and other factors related to surgery may influence the results.

 Other comparative reviews show few differences between the techniques. Several factors related to patients, indications and technique are involved and it is difficult to understand the importance of each. Furthermore preference, training and experience of each surgeon still overlaps the data from the studies. Even with great care to obtain and evaluate data such studies may have little influence on surgeons' treatment decisions.

I have read this submission. I believe that I have an appropriate level of expertise to confirm that it is of an acceptable scientific standard.

# Peer review: Referee response for version 1

**DOI:** 10.5256/f1000research.9699.r16070

**Published:** 2016-09-21

**Authors:** Terje Sundstrøm, Morten Lund-Johansen

**Affiliations:** 1 Department of Neurosurgery, Haukeland University Hospital, Bergen, Norway

The authors have designed a study to investigate the difference in outcome between microdiscectomy and open discectomy as treatments for lumbar disc herniation. Microdiscectomy refers to a discectomy with the use of magnification (microscope or loupes) and open discectomy refers to a discectomy without the use of magnification. This is a real-world, large, observational study using data from 2006 to 2014 from the national spine registry in Norway. The study is well designed, will have a high degree of external validity and can approximate the strength of a randomized controlled trial; the authors will include propensity score matching to reduce the potential bias of a range of baseline co-variables.

 Previous retrospective and prospective observational studies of microdiscectomy versus open discectomy have landed on both sides of the fence. However, three randomized controlled trials have not demonstrated any superiority between the two operative techniques ^1^^-^^3^.

 In an updated Cochrane review from 2007 ^4^, it was argued that microdiscectomy and open discectomy are both gold standard treatments for symptomatic lumbar disc herniation. Nonetheless, contemporary use of microdiscectomy or open discectomy is probably relatively independent of the accumulated scientific evidence, and to a larger extent reliant on the training and expertise of the surgeon, as well as the availability and quality of visual augmentation systems. We feel that this study is a little like documenting the need for binoculars in birdwatching. An experienced birdwatcher may identify nearly every species without binoculars, but for some reason they all use binoculars; they can simply see much better. We can agree that there is still a need for more and better evidence on the relative clinical outcomes and cost-effectiveness of microdiscectomy versus open discectomy, but microscopes significantly augment vision and – let us not forget – greatly facilitate teaching. For these latter reasons, most (if not all) major spine units worldwide utilize some kind of magnification.

 It would be interesting to know if the relative number of microdiscectomies and open discectomies varied over time, or if its simply a gradual conversion from open surgery to microsurgery. Whatever the outcome of this study may be, we are concerned about the interpretation, because we do not know why some surgeons chose not to use any kind of visual magnification. In the study period in Norway, most of the open discectomies were probably done by experienced surgeons (and some by reckless individuals), whereas many of the microdiscectomies were probably done by less experienced surgeons or surgeons in training. Including the identity of the surgeons in the data analysis could help unmask possible differences more attributable to the surgeon than the surgical technique *per se*.

 Visual augmentation devices are widely used, and microscopes are unsurpassable in the training of young surgeons. From this, and the accumulated literature in this field showing similar or minor differences, it is in our opinion unlikely that any positive or negative result of microdiscectomy will affect the way surgeons choose to operate lumbar disc herniations.

We have read this submission. We believe that we have an appropriate level of expertise to confirm that it is of an acceptable scientific standard.

## References

[1] TullbergTIsacsonJWeidenhielmL: Does microscopic removal of lumbar disc herniation lead to better results than the standard procedure? Results of a one-year randomized study. *Spine (Phila Pa 1976)*.1993;18(1) :24-7 843432110.1097/00007632-199301000-00005

[2] HenriksenLSchmidtKEskesenVJantzenE: A controlled study of microsurgical versus standard lumbar discectomy. *Br J Neurosurg*.1996;10(3) :289-93 879954110.1080/02688699650040160

[3] KatayamaYMatsuyamaYYoshiharaHSakaiYNakamuraHNakashimaSItoZIshiguroN: Comparison of surgical outcomes between macro discectomy and micro discectomy for lumbar disc herniation: a prospective randomized study with surgery performed by the same spine surgeon. *J Spinal Disord Tech*.2006;19(5) : 10.1097/01.bsd.0000211201.93125.1c 344-7 10.1097/01.bsd.0000211201.93125.1c 16826006

[4] GibsonJNWaddellG: Surgical interventions for lumbar disc prolapse: updated Cochrane Review. *Spine (Phila Pa 1976)*.2007;32(16) : 10.1097/BRS.0b013e3180bc2431 1735-47 10.1097/BRS.0b013e3180bc2431 17632394

# Response

**Published:** 2016-09-26

**Authors:** Andreas Sørlie

**Affiliations:** Department of Neurosurgery, Unviersityhospital of North-Norway, Tromsø , Norway

Thank you for the referee report.

 We appreciate your concern regarding the interpretation of the results. One challenge in this regard is that the different centers have different approaches according to surgeon preference, and that the technique used might be the best (whichever technique is used) in the hands of that particular surgeon. However, we believe the results might influence both the chosen approach by the particular surgeon as well as the preferred handling of the group of patients by a department, as well as for training of future surgeons. Some departments in Norway perform both microdiscectomy as well as open discectomy.

 There seems to be a slight shift towards microdiscectomy over time, but several open discectomies are still performed every year in Norway. We can include this data in the discussion when the results are ready.

 Andreas Sørlie
